# Long-term stability of clopidogrel solid dispersions—Importance of *in vitro* dissolution test

**DOI:** 10.1371/journal.pone.0266237

**Published:** 2022-04-04

**Authors:** Ehlimana Osmanović Omerdić, Larisa Alagić-Džambić, Marko Krstić, Maja Pašić-Kulenović, Đorđe Medarević, Branka Ivković, Dragana Vasiljević

**Affiliations:** 1 Development and Registration Department, Bosnalijek d.d., Sarajevo, Bosnia and Herzegovina; 2 Quality Assurance and Quality Control Department, Bosnalijek d.d., Sarajevo, Bosnia and Herzegovina; 3 Department of Analytical Chemistry, University of Belgrade—Faculty of Pharmacy, Belgrade, Serbia; 4 Department of Pharmaceutical Technology and Cosmetology, University of Belgrade—Faculty of Pharmacy, Belgrade, Serbia; 5 Department of Pharmaceutical Chemistry, University of Belgrade—Faculty of Pharmacy, Belgrade, Serbia; University of Alcalá, SPAIN

## Abstract

Formulation of solid dispersions (SDs), in which the drug substance is dissolved or dispersed inside a polymer matrix, is one of the modern approaches to increase the solubility and dissolution rate of poorly soluble active pharmaceutical ingredients (APIs), such as clopidogrel. In the form of a free base, clopidogrel is unstable under increased both high moisture and temperature, so it is most often used as its salt form, clopidogrel hydrogen sulfate (CHS).The aim of this study was the formulation, characterization, and long-term stability investigation of CHS solid dispersions, prepared with four different hydrophilic polymers (poloxamer 407, macrogol 6000, povidone, copovidone) in five API/polymer ratios (1:1, 1:2, 1:3, 1:5, 1:9). SDs were prepared by the solvent evaporation method, employing ethanol (96% v/v) as a solvent. Initial results of the *in vitro* dissolution test showed an increase in the amount of dissolved CHS from all prepared SD samples compared to pure CHS, corresponding physical mixtures (PMs), and commercial tablets. SDs, prepared with poloxamer 407, macrogol 6000, and copovidone, at CHS/polymer ratios 1:5 and 1:9, notably increased the amount of dissolved CHS (> 80%, after 60 min), thus they were selected for further characterization. To assess the SDs long-term stability, *in vitro* dissolution studies, clopidogrel content determination, differential scanning calorimetry (DSC), and Fourier transform infrared spectroscopy (FT-IR) were performed initially and after 12 months of long-term stability studies under controlled conditions (25°C, 60% RH) meeting the ICH guideline Q1A (R2) requirements. The clopidogrel content in the selected samples was very similar at the beginning (96.13% to 99.93%) and at the end (95.98% to 99.86%) of the conducted test. DSC curves and FT-IR spectra of all SD samples after 12 months of stability study, showed the absence of CHS crystallization, which is an indication of good stability. However, the *in vitro* dissolution test showed a considerable reduction in CHS released from SDs with macrogol 6000. The amount of dissolved CHS from SDs with macrogol 6000 was initially 94.02% and 92.01%, and after 12 months of stability study, only 65.13% and 49.62%. In contrast, the amount of dissolved CHS from SDs prepared with poloxamer 407 and copovidone was very similar after 12 months of the stability study compared to the initial values. Results obtained indicated the great importance of the in vitro dissolution test in determining the long-term stability and quality of SDs.

## 1. Introduction

A number of formulation strategies have focused on increasing solubility and dissolution rate with the aim to improve the oral bioavailability of poorly soluble drugs like clopidogrel. One of the most promising is solid dispersions (SDs) formulation. SDs can be defined as a dispersion of one or more active pharmaceutical ingredients (APIs) in an inert carrier or matrix in the solid-state prepared by melting method, solvent evaporation method, or combination of these two methods [1]. SDs can be prepared using cost-effective manufacturing technologies with well-known, widely used, safe excipients [2]. Mechanisms of enhancement solubility and dissolution rate of API in SDs include particle size reduction, formation of solid solutions of drug in an inert polymer matrix, enhanced porosity of SDs, improved wettability, solubilization of API, or conversion of drug from crystalline into the amorphous state [3, 4]. It was recently reported that between 2007 and 2017, the US Food and Drug Administration approved 19 commercial SDs products, mainly in tablets and capsules dosage forms [5]. The increase in the number of approved and commercially available products gives additional importance to more substantial research of SDs. Due to its complexity, there are numerous challenges regarding of the physicochemical properties of used API and polymers, and therefore influencing on the stability of SDs and formulation of the final dosage form. It has been reported that stability, which is one of the main issue of SDs, can be successfully resolved by the use of appropriate polymer and preparation method [2, 6, 7].

Clopidogrel, methyl (+) -(S)-α-(2-chlorophenyl)-6,7-dihydrothieno[3,2-c] pyridine-5(4H)-acetate, is an inhibitor of platelet activation and aggregation through the irreversible binding of its active metabolite to the P_2_Y_12_ class of ADP receptors on platelets. The inhibiting activity makes clopidogrel an effective API for reducing the induce of ischemic strokes, myocardial infarction, and vascular disease [8, 9]. In a free base form, clopidogrel is an oily substance with high viscosity, unstable under increased moisture and temperature, so salt forms are commonly used. The most commonly used salt is clopidogrel hydrogen sulfate (CHS) (syn. clopidogrel bisulfate) [10]. CHS belongs to BCS class II APIs with low solubility that limits its oral bioavailability and therapeutic effectiveness. CHS has been found to exist in six polymorphic forms and an amorphous form. Still, only polymorphic forms I and II are used in the formulation of dosage forms due to better stability and bioavailability compared to other forms [10–12]. Form II has a melting point range between 176–178°C, is thermodynamically more stable, and has better compactibility than form I, and therefore is more acceptable for the manufacturing process [12–14].

The aim of this study was the formulation, characterization, and long-term stability evaluation of CHS solid dispersions, prepared with four different hydrophilic polymers (poloxamer 407, macrogol 6000, povidone, copovidone) in five API/polymer ratios (1:1, 1:2, 1:3, 1:5, 1:9).

## 2. Materials and methods

### 2.1. Materials

In this study, clopidogrel hydrogensulfate (Ph. Eur. 10.0) was used as a model API. Four different hydrophilic polymers: poloxamer 407 (Kolliphor^®^ P407, BASF, Germany), macrogol 6000 (Polyglykol 6000 S, Clariant Export AG, Switzerland), povidone (Kollidon^®^ 30, BASF, Germany), and copovidone (Kollidon^®^ VA 64, BASF, Germany), were used as carriers. Ethanol 96% (v/v) was used as a solvent for SDs preparation by solvent evaporation method. All excipients were of pharmaceutical grade.

Other reagents and chemicals used for the characterization of SDs were of analytical grade. API, excipients, and other reagents and chemicals were kindly provided as a gift from Bosnalijek d.d. (Sarajevo, Bosnia and Herzegovina).

### 2.2. Methods

#### 2.2.1. Preparation of solid dispersions and physical mixtures

CHS solid dispersions were prepared by the standard solvent evaporation method, employing ethanol (96% v/v) as a solvent. The composition of prepared SDs is presented in Table 1. The required quantities of CHS and polymer were dissolved in the corresponding amount of solvent to get a clear solution. The solvent was then removed by evaporation under reduced pressure (vacuum) on a rotary vacuum evaporator (Rotavapor^®^ R-205, Büchi, Switzerland) at a temperature of 55°C with constant mixing at 95 rpm. The resulting solid mass was gentle ground in a mortar with a pestle to obtain powder form and shifted through a sieve 500 μm (Ph.Eur.). Prepared SDs were filled and stored in a sealed amber glass bottle with a polyethylene screw.

**Table 1:** Composition of CHS solid dispersions and corresponding physical mixtures.

No. of formulation	Formulation	Polymer	CHS/ polymer ratio	Physical mixure
**1**	P1	Poloxamer 407	1:1	PPM1
**2**	P2	Poloxamer 407	1:2	PPM2
**3**	P3	Poloxamer 407	1:3	PPM3
**4**	P5	Poloxamer 407	1:5	PPM5
**5**	P9	Poloxamer 407	1:9	PPM9
**6**	M1	Macrogol 6000	1:1	MPM1
**7**	M2	Macrogol 6000	1:2	MPM2
**8**	M3	Macrogol 6000	1:3	MPM3
**9**	M5	Macrogol 6000	1:5	MPM5
**10**	M9	Macrogol 6000	1:9	MPM9
**11**	K1	Povidone	1:1	KPM1
**12**	K2	Povidone	1:2	KPM2
**13**	K3	Povidone	1:3	KPM3
**14**	K5	Povidone	1:5	KPM5
**15**	K9	Povidone	1:9	KPM9
**16**	C1	Copovidone	1:1	CPM1
**17**	C2	Copovidone	1:2	CPM2
**18**	C3	Copovidone	1:3	CPM3
**19**	C5	Copovidone	1:5	CPM5
**20**	C9	Copovidone	1:9	CPM9

The physical mixtures (PMs) of CHS and polymers (Table 1) were prepared by mixing the API and polymer using mortar and pestle. Obtained mixtures were shifted through sieve 500 (Ph. Eur), filled, and stored in a sealed amber glass bottle with a polyethylene screw.

For *in vitro* dissolution studies, SDs and PMs were filled into hard gelatin capsules which contained a therapeutic dose of clopidogrel (75 mg) and stored in a sealed amber glass bottle with a polyethylene screw until analyses were performed.

#### 2.2.2. *In vitro* dissolution studies

*In vitro* dissolution studies were performed using USP Apparatus 1 (basket), rotation speed of 75 rpm, in 900 mL of phosphate buffer (pH 6.8) at 37°C ± 0.5°C. Tests were conducted with a Vankel VK 7010 dissolution bath and a Varian fraction sample collection module (Agilent, USA).

Aliquots of 1.5 mL were removed at predetermined time points (15, 30, 45, and 60 min) and replaced with an equal volume of fresh dissolution medium. Samples were filtered through a 35 μm filter (Micron Full Flow Filter, Agilent, USA) and collected directly into an HPLC vial. The CHS concentration was determined by the RP-HPLC method, as described in the previously published paper [15]. *In vitro* dissolution studies were performed in triplicate, and the data are expressed as the mean value ± standard deviation. The obtained dissolution profiles were fitted into different empirical models, and the corresponding correlation coefficients (R^2^) were calculated.

#### 2.2.3. Determination of clopidogrel content

Accurately weighed quantity of selected SDs, equivalent to 75 mg of clopidogrel, was diluted with the mobile phase to a volume of 100 ml. 1 ml of this filtered solution was further diluted with 10 ml mobile phase. The content of clopidogrel was determined by the mentioned RP-HPLC method [15].

#### 2.2.4. Differential scanning calorimetry (DSC)

DSC analyses of API, polymers, and selected SDs were performed using a DSC1 instrument (Mettler Toledo, Giessen, Germany). A precisely measured amount of sample (1–5 mg) was placed into pierced 40 μl aluminium pans, and subjected to heating from 20 to 250°C with a heating rate of 10°C/min using nitrogen as purge gas at a flow rate of 50 ml/min.

#### 2.2.5. Fourier transform infrared spectroscopy (FT-IR)

FT-IR spectra of API, polymers, and selected SDs were acquired using Nicolet iS10 (Thermo Scientific, Waltham, USA) FT-IR spectrometer, equipped with a single reflection ATR system (Smart iTR, Thermo Scientific, Waltham, USA) with diamond plate and Zn-Se lens. The samples were directly kept on the sample holder, and spectra were recorded as an average of 16 scans in the frequency range from 4000 to 650 cm^-1^, with a resolution of 4 cm^-1^. Sixteen recordings were made for each sample and then averaged to obtain a spectrum.

#### 2.2.6. Long-term stability studies

Selected SDs, filled into sealed amber glass bottles with a polyethylene screw, were stored for 12 months in chambers for stability testing (Pharma 2000, Weiss Technik, Germany) under the conditions of 25°C ± 2°C and 60% RH ± 5% RH, according to ICH guideline Q1A (R2) [16]. To assess the long-term stability of SDs, *in vitro* dissolution studies, clopidogrel content, DSC, and FT-IR were performed initially and after 12 months of stability studies under mentioned conditions.

## 3. Results and discussions

In this study, 20 samples of clopidogrel hydrogensulfate SDs were prepared, with different hydrophilic polymers (poloxamer 407, macrogol 6000, povidone, and copovidone) and different API/polymer ratios (1:1, 1:2, 1:3, 1:5, and 1:9). Initially, an *in vitro* dissolution test was performed. This test is an easy, fast, inexpensive method, which during research and development may show differences in the dissolution rate of API from the tested samples. Only samples from which at least 80% CHS was dissolved after 60 min were selected for further characterization.

### 3.1. *In vitro* dissolution studies

Firstly, an *in vitro* dissolution test was performed with 20 prepared SDs, corresponding PMs, pure CHS, and commercial immediate release film coated tablet.

**Fig 1:**
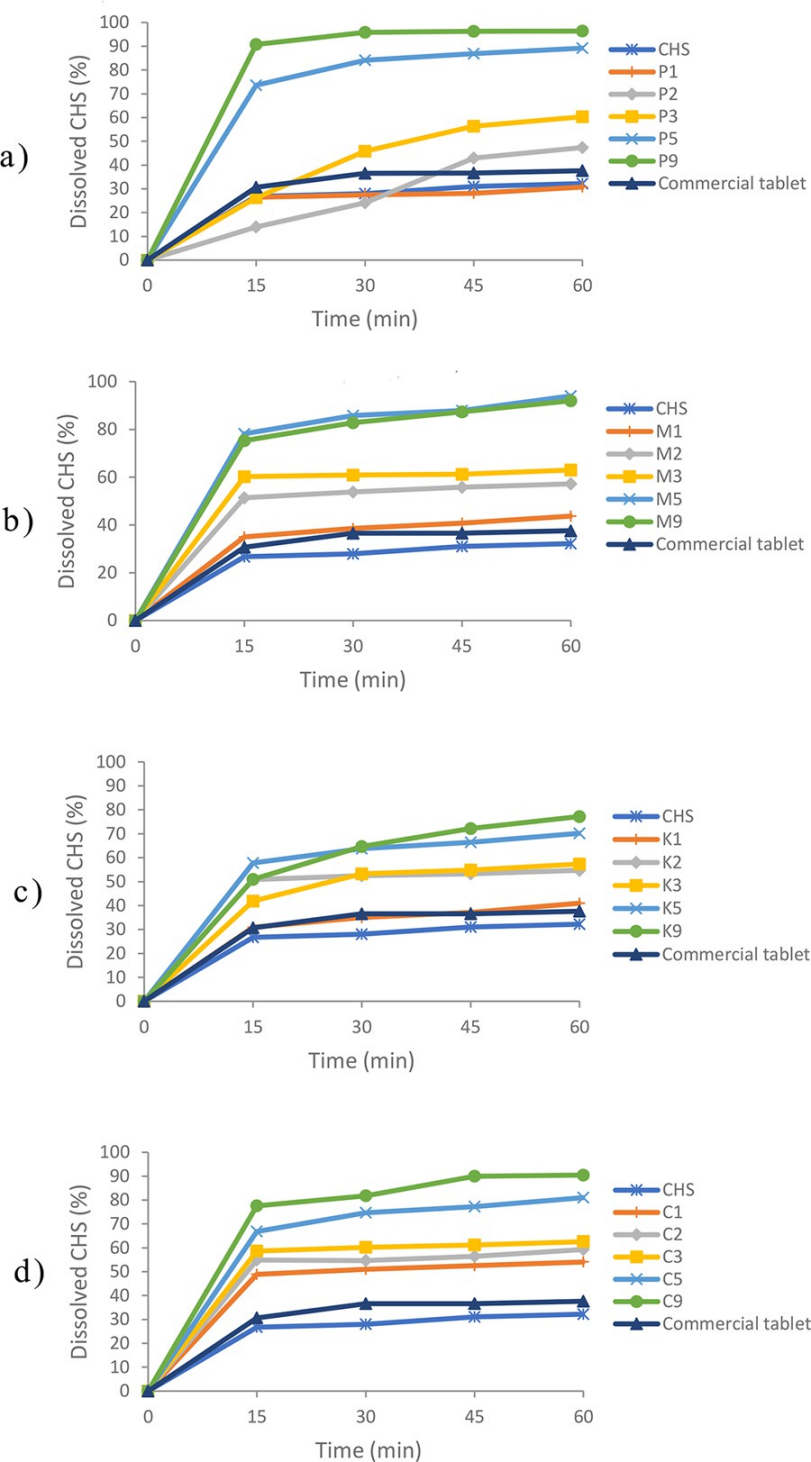
Comparative *in vitro* dissolution profiles of pure CHS, SDs and commercial tablet. a) SDs with poloxamer 407 b) SDs with macrogol 6000 c) SDs with povidone d) SDs with copovidone.

Dissolution profiles of pure CHS, SDs, and commercial tablet are presented in (Fig 1A–1D). After 60 min, the amount of dissolved CHS was between 30.74% and 96.38% for SDs (Table 2), 32.20% for pure CHS, and 37.62% for commercial tablet. Except in the case of sample P1, the amount of dissolved CHS from SDs was notably higher than the corresponding PMs (Table 2), pure CHS, and commercial tablet. Sample P1 contained the least amount of polymer (CHS/poloxamer 407 ratio 1:1), so it can be assumed that in this case, the amount of poloxamer 407 was not sufficient to increase the solubility and dissolution rate of CHS. In other samples, it could be assumed that SDs increased the amount of dissolved CHS due to the transition of the crystalline form of API to amorphous, reducing particle size, enhanced wettability, and polymers’ ability to solubilize API, as mentioned earlier in literature [17–20].

**Table 2:** The amount of dissolved CHS after 60 min from SDs and PMs.

Solid dispersion	Dissolved CHS, % (S.D.)	Physical mixture	Dissolved CHS, % (S.D.)
**P1**	30.74 (0.68)	PPM1	29.60 (0.58)
**P2**	47.38 (1.00)	PPM2	32.60 (2.04)
**P3**	60.34 (0.98)	PPM3	34.94 (0.51)
**P5**	89.21 (1.86)	PPM5	42.71 (1.04)
**P9**	96.38 (1.68)	PPM9	42.71 (1.21)
**M1**	43.72 (1.50)	MPM1	27.34 (0.87)
**M2**	57.22 (1.01)	MPM2	30.83 (0.53)
**M3**	63.01 (1.51)	MPM3	36.06 (0.96)
**M5**	94.03 (0.85)	MPM5	45.20 (1.40)
**M9**	92.01 (1.60)	MPM9	50.45 (2.14)
**K1**	40.98 (0.32)	KPM1	22.31 (0.93)
**K2**	54.75 (0.71)	KPM2	23.07 (2.13)
**K3**	57.34 (2.34)	KPM3	25.30 (1.98)
**K5**	70.20 (0.55)	KPM5	27.04 (0.10)
**K9**	77.22 (2.58)	KPM9	29.91 (3.06)
**C1**	54.05 (0.61)	CPM1	30.93 (1.28)
**C2**	59.28 (1.06)	CPM2	31.86 (0.54)
**C3**	62.56 (0.35)	CPM3	33.22 (1.10)
**C5**	81.01 (0.93)	CPM5	28.77 (3.41)
**C9**	90.44 (1.33)	CPM9	44.35 (0.53)

S.D. standard deviation.

In the case of SDs prepared with poloxamer 407, povidone, and copovidone, with the increase in the content of the polymer in the SDs, there was an increase in the amount of dissolved CHS. However, in macrogol SDs, the larger amount of CHS (94.03%) was dissolved from the sample M5, which had a CHS/polymer ratio of 1:5. The dissolved CHS amount from the sample with a CHS/macrogol 6000 ratio of 1:9 (M9) was 92.01%. This could be explained by the assumption that in the sample M9 around the CHS particles, a viscous polymer layer was formed, which prevent further dissolution improvement [21].

Based on the obtained results, it could be concluded that SDs, prepared with three different polymers (poloxamer 407, macrogol 6000, and copovidone), at CHS/polymer ratios 1:5 and 1:9, notably increase the amount of dissolved CHS. SDs in which the amount of dissolved CHS after 60 min was greater than 80% (P5, P9, M5, M9, C5, and C9) were selected for further characterization. In the case of SDs prepared with povidone (K1 –K9), the amount of dissolved CHS after 60 min was from 40.98% to 77.22%. These samples did not meet the stated requirement and were excluded from further study.

### 3.2. Determination of clopidogrel content

Initially, clopidogrel content in the selected SDs was in the range of 96.13% to 99.93% (Table 3). These results were in line with the requirements of the guideline “Specifications and Control Tests on the Finished Product-3AQ11a”, where maximum acceptable deviations in the APIs content should not exceed ± 5% [22].

**Table 3:** Clopidogrel content in selected SDs (initially, and after 12 months of stability studies).

SDs	Clopidogrel content (%)	Clopidogrel content (%)
	0 months	12 months
**P5**	99.93	99.86
**P9**	99.91	99.38
**M5**	98.99	98.51
**M9**	96.13	97.41
**C5**	96.77	95.82
**C9**	96.35	95.98

### 3.3. Differential scanning calorimetry (DSC)

**Fig 2:**
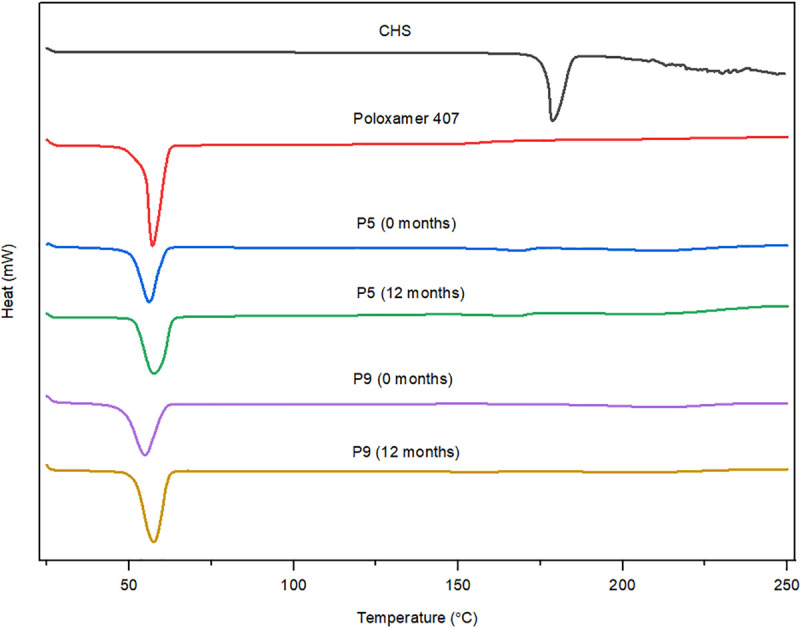
DSC curves of CHS, poloxamer 407, and SDs with poloxamer 407 (P5 and P9) initially, and after 12 months of stability studies.

**Fig 3:**
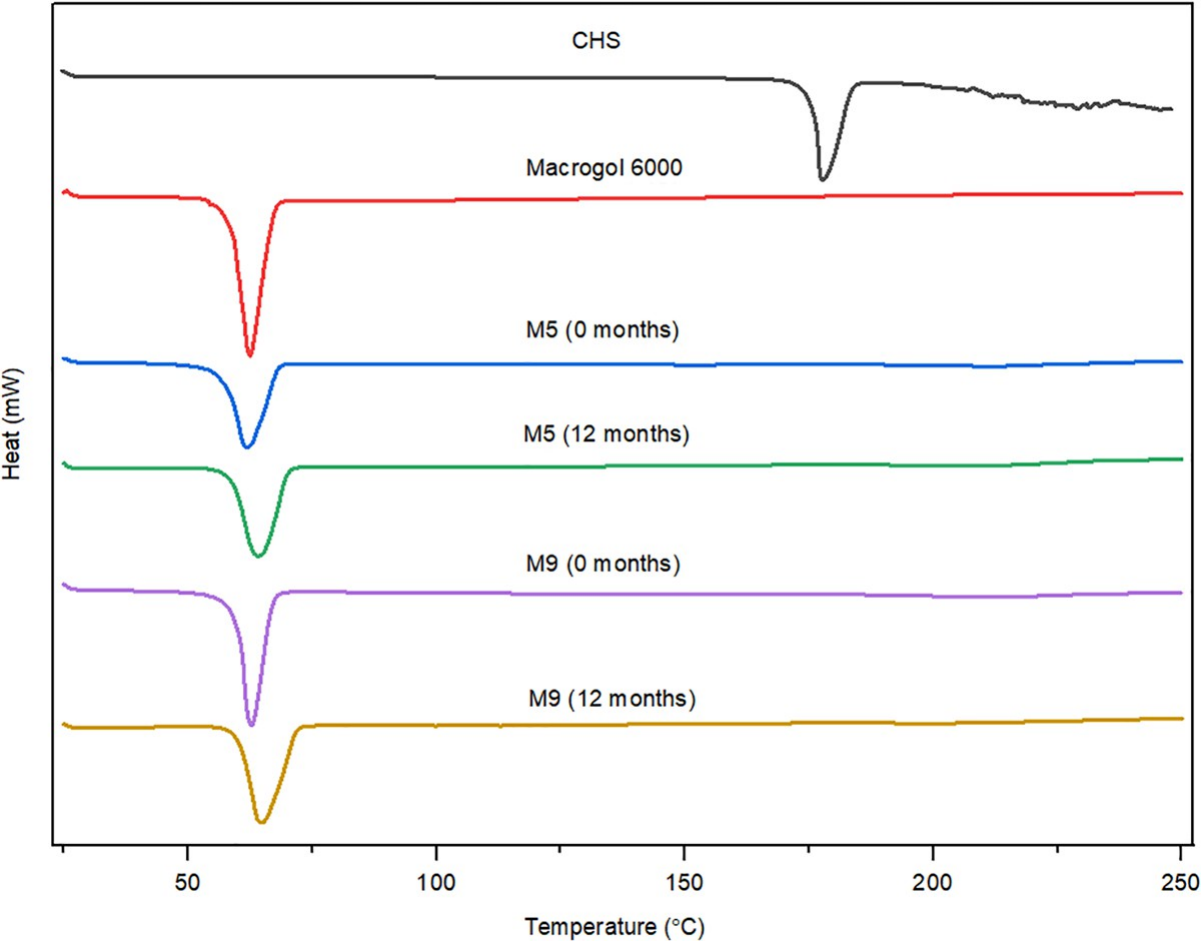
DSC curves of CHS, macrogol 6000, and SDs with macrogol 6000 (M5 and M9) initially, and after 12 months of stability studies.

**Fig 4:**
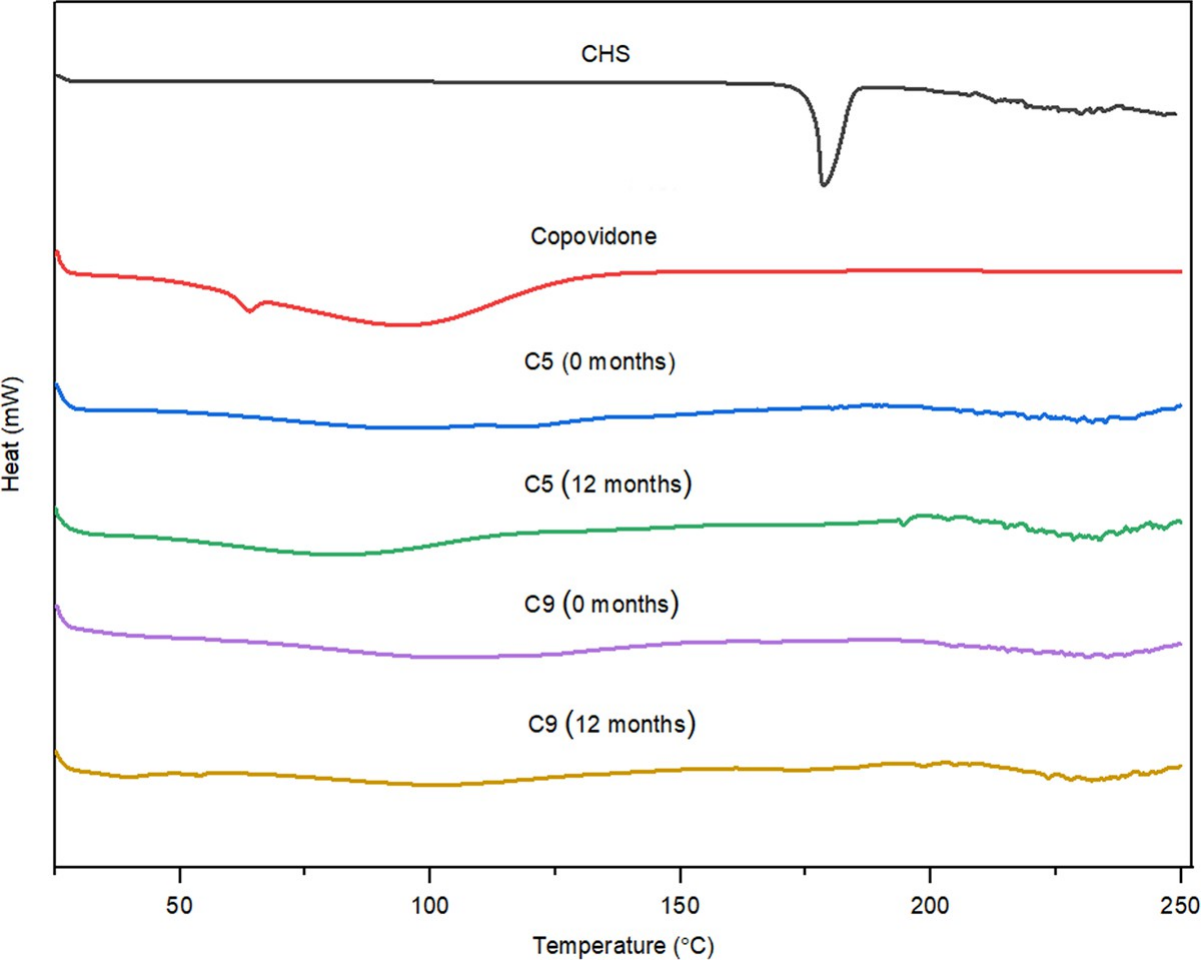
DSC curves of CHS, copovidone, and SDs with copovidone (C5 and C9) initially, and after 12 months of stability studies.

**Fig 5:**
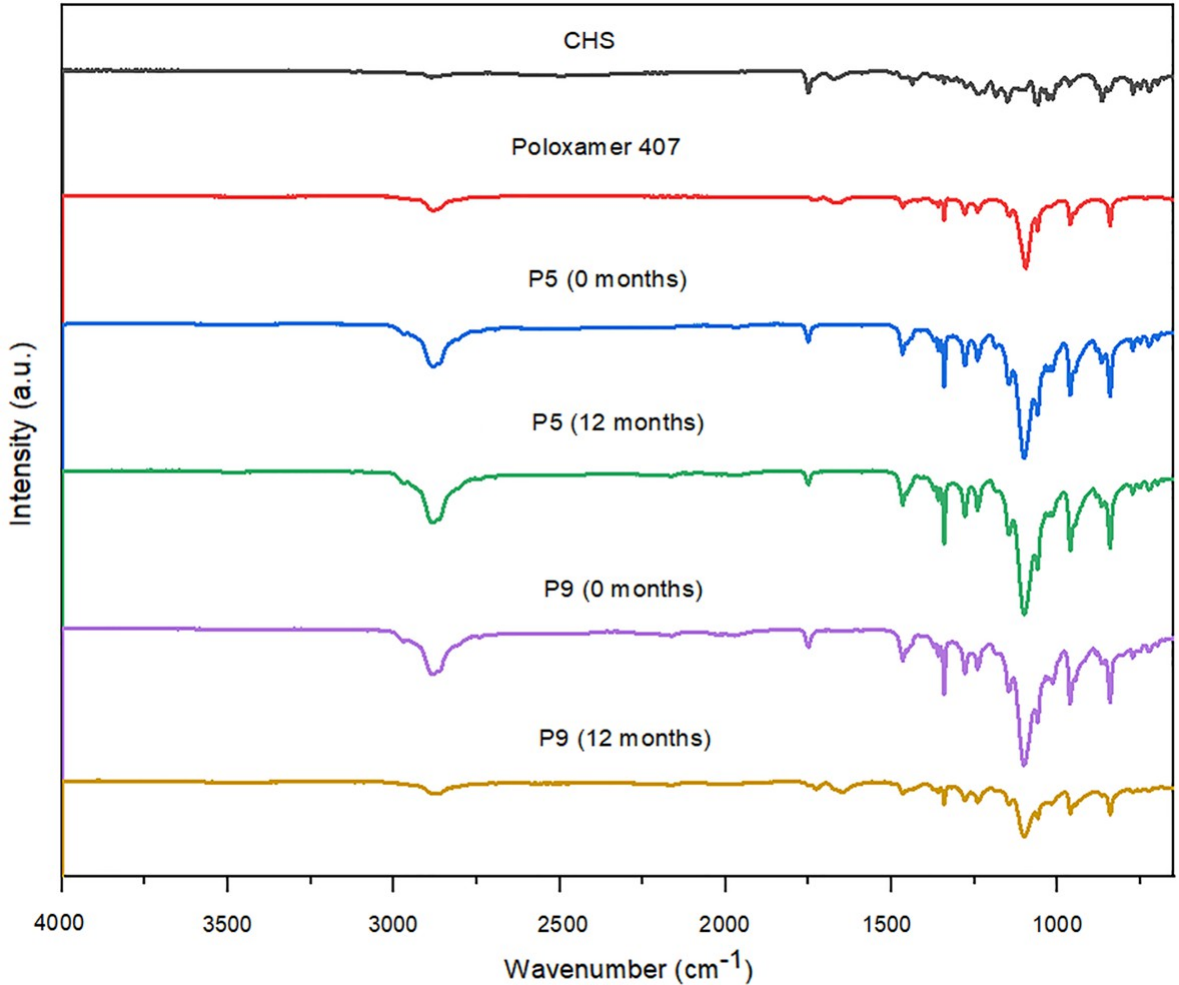
FT-IR spectra of CHS, poloxamer 407, and SDs with poloxamer 407 (P5 and P9) initially, and after 12 months of stability studies.

**Fig 6:**
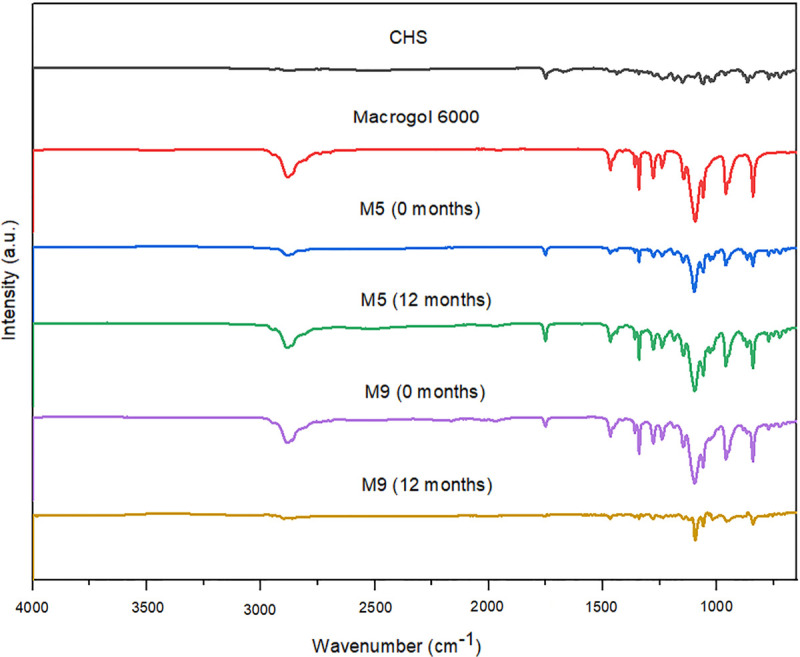
FT-IR spectra of CHS, macrogol 6000, and SDs with macrogol 6000 (M5 and M9) initially, and after 12 months of stability studies.

**Fig 7:**
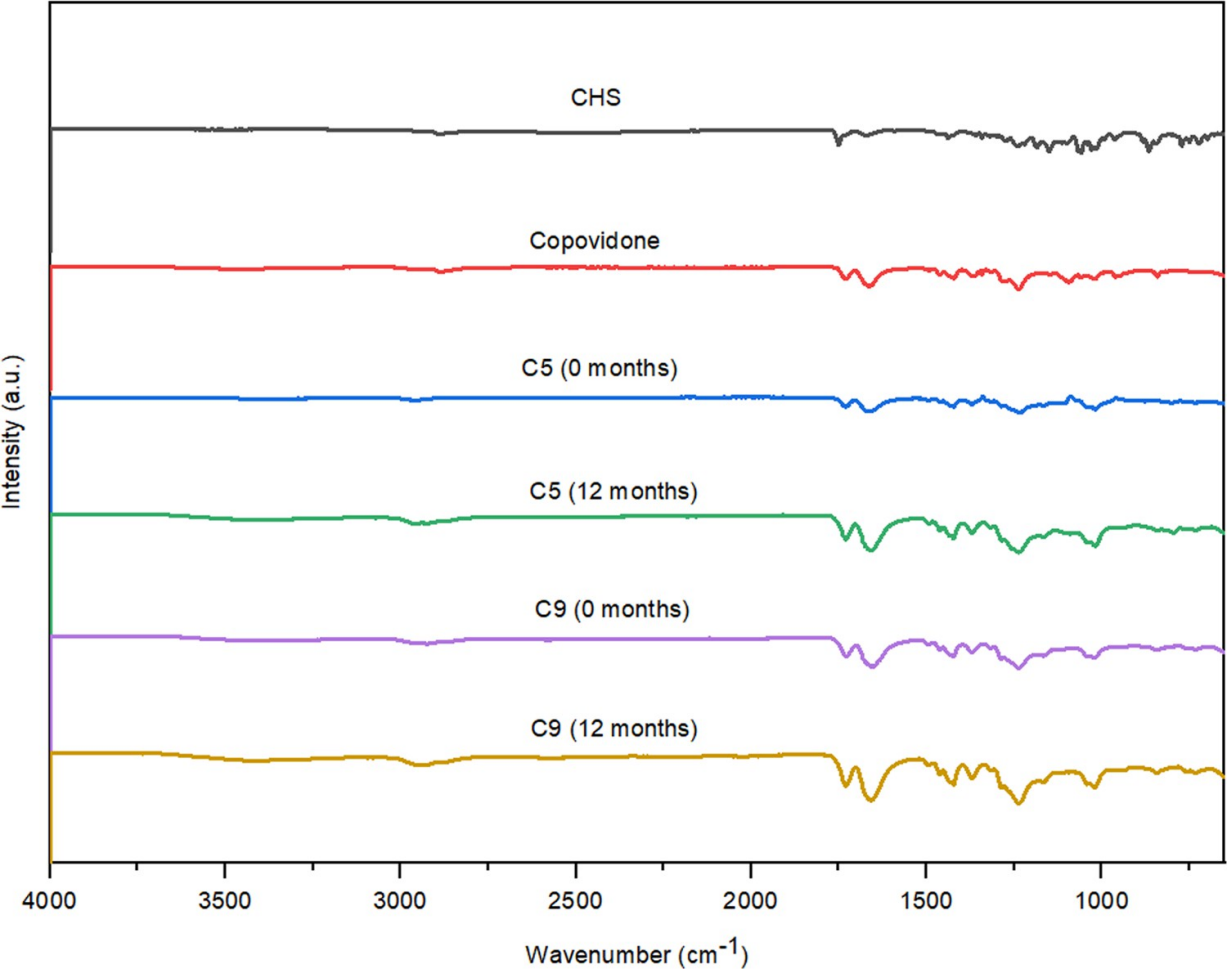
FT-IR spectra of CHS, copovidone, and SDs with copovidone (C5 and C9) initially, and after 12 months of stability studies.

DSC has been used to measure the melting point of API and polymer, API-polymer miscibility, polymorphic form transformation, a crystallization tendency of an API, and molecular mobility [23]. In the SDs, if the drug is in amorphous form, no endothermic peak will be present on the DSC curve. Figs 2–4 illustrate the DSC curves of pure CHS, polymers, and selected SDs (initially and after 12 months of stability studies). A small number of thermal events were observed in all the DSC curves. DSC curve of pure CHS showed a single endothermic peak at a temperature of 178°C, corresponding to the melting point of the CHS. This indicated that CHS was in polymorphic form II, which is in accordance with the literary data of melting point range between 176–178°C [10, 12, 13]. Polymorphic form I of CHS has a melting point at slightly higher temperatures, 198–200°C [10, 12, 13]. Polymorphic form II of CHS is the most stable form at ambient conditions, and it is also used more often in commercial dosage forms (tablets and film-coated tablets) [14]. DSC curves of polymers showed an endothermic peak for poloxamer 407 at 57°C (Fig 5) and macrogol 6000 at 63°C (Fig 6), corresponding to the melting points of these polymers [11, 21, 24]. Broad endotherms of copovidone were observed at 63°C and 100–107°C, respectively (Fig 7) [19, 25], which can result from combination of several thermal events, such as glass transition, evaporation of absorbed water and polymer relaxation. Characteristic peaks for the melting point of crystalline CHS in all SDs curves wholly disappeared, which was indicated that most of the API was existed in the amorphous state. Peaks that correspond to poloxamer 407 and macrogol 6000 in SDs curves were remained in close positions as in pure polymers. Copovidone endotherms were further stretched or almost completely disappeared in SDs curves. Small endothermic peaks seen on DSC curves of API and SDs after the melting temperature of CHS were most likely caused by the thermally induced degradation of CHS [12].

### 3.4. Fourier transform infrared spectroscopy (FT-IR)

FT-IR spectroscopy was used to examine interactions between the CHS and the polymers in solid dispersions. Intermolecular interactions between API and polymer are essential in stabilizing API amorphous form in the SDs matrix. These interactions also play the most crucial role in preventing the crystallization of API [26, 27].

The FT-IR spectra of pure CHS, polymers, and selected SDs (initially and after 12 months of stability studies) are presented in Figs 5–7. The FT-IR spectrum of CHS shows characteristic bands at 3121 cm^–1^ (aromatic CH vibrations), due to the presence of chlorophenyl ring, 1750 cm^-1^ with higher intensity, due to the presence of the ester functional group (C = O stretching vibrations), 1437 cm^-1^ (N-H deformations), 1186 cm^-1^ (in-plane motion of the C-H) and 1150 cm^-1^ (rocking of CH_3_) and 1028 cm^-1^ (pyridine-methylene rock) which is in line with the spectra previously reported in the literature [12, 13, 28].

The SDs spectra looked very similar to the spectra of individual components, CHS and polymers, with minimal differences. For SDs with poloxamer 407 (P5 and P9) and macrogol 6000 (M5 and M9) the differences between observed spectra for most characteristic band is about 1–5 cm^-1^. Shifting of the peak from 1150 to 1147 cm^-1^ were observed on these spectra. These subtle changes probably resulted in hydrogen bonds between CHS and polymers. For SDs with copovidone (C5 and C9) absorption band at 1750 cm^-1^ were observed at 1730–1731 cm^-1^ and CHS band positioned at 1150 cm^-1^ shifted to 1166 cm^-1^. For C5 solid dispersion CHS band at 1186 and 1028 cm^-1^ shifted to 1181 and 1034 cm^-1^, respectively, and for C9 same bands shifted to 1184 and 1042 cm^-1^, respectively. These observed changes in FTIR spectra of SDs could indicate the existence of intermolecular interactions between CHS and polymers. Physical interactions between molecules can be held responsible for reducing the level of CHS crystallinity and improving CHS dissolution [24, 29]. However, no additional peaks were observed in the FT-IR spectra of SDs, indicating the absence of any chemical interaction.

### 3.5. Long-term stability studies

**Fig 8:**
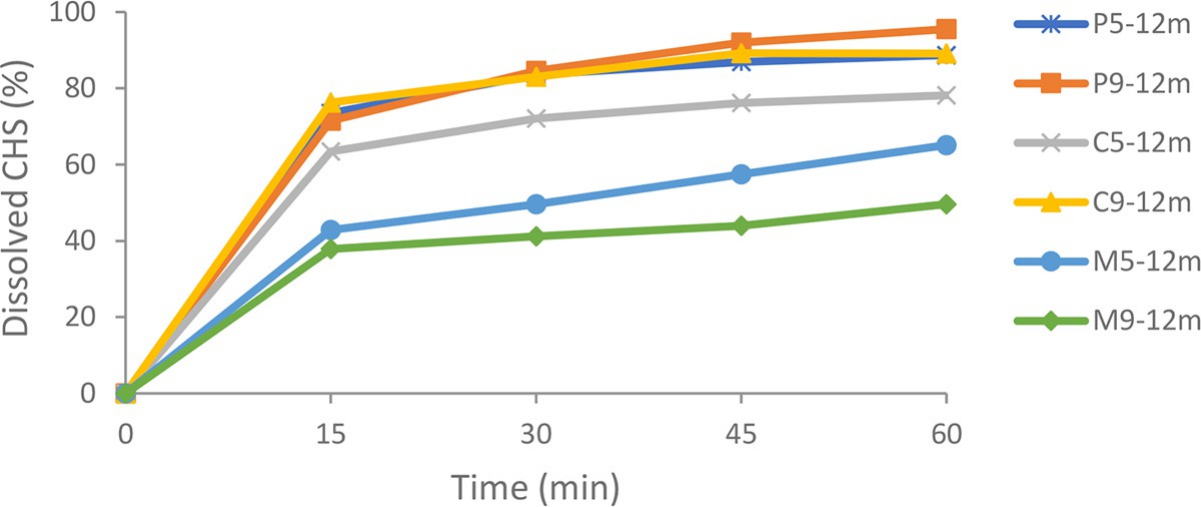
Comparative dissolution profiles of selected SDs after 12 months of stability studies.

Physical stability is the main issue for the development SDs in a final dosage form. Long-term stability of SDs is necessary to ensure an acceptable shelf-life of the final dosage form. The optimal type of polymer and API loading, good miscibility between API and polymer, and enhancing drug-polymer interaction have proven to be a good strategy for improving the stability of SDs [30]. After 12 months of stability studies, selected SDs (P5, P9, C5, C9, M5, and M9) were analyzed using *in vitro* dissolution tests, clopidogrel content, DSC, and FT-IR, and were compared to initial results. The amount of dissolved CHS from SDs with poloxamer 407 and copovidone after 12 months of stability studies were very similar to initial results (Figs 1A–1C, and 8). In the case of SDs with macrogol 6000 (M5 and M9), after 12 months of stability studies, a decrease in the amount of dissolved CHS was observed (65.13% and 49.62%, respectively) compared to the initial values (94.02% and 92.01%, respectively) (Figs 1C and 8). This could be explained by changing the physical properties of the polymer during storage. Damian et al. [31] showed that in SD with an antiviral drug UC-781 and hydrophilic polymer macrogol 6000, an increase in enthalpy of fusion was observed during stability studies, especially expressed at a temperature of 25°C. The absence of crystallinity of the UC-781 in the SDs with macrogol 6000 initially and after 12 months of stability studies was confirmed by DSC and X-ray powder diffraction. It was assumed that reason for decreasing in UC-781 dissolution was the reorganization in the crystalline structure of the polymer that occurred during storage, which led to a decrease in the dissolution rate of API from the SDs. A similar decrease in the dissolution of API from SDs with macrogol 6000 after stability studies were also reported by Ford and Rubinstein [32, 33]. When compared SDs with macrogol 6000, for M9 was noticed a higher decrease in the amount of dissolved API, probably because of a higher amount of polymer in the formulation.

To describe CHS release mechanism from SDs (initially and after 12 months of stability studies), mathematical models such as zero-order, first-order, Korsmeyer-Peppas, Higuchi, and Hixson-Crowell models were used. Initially, all selected SDs had the greatest R^2^ in the Korsmeyer-Peppas model (Table 4). According to this model, the drug was transported via Fickian diffusion when the parameter n was below or equal to 0.45. In contrast, the n value between 0.45 and 0.89 indicated anomalous transport (non-Fickian diffusion). Case I transport is defined as a zero-order release model when n value was equal to 0.89, while n values were above 0.89, suggesting a super case II transport [34, 35]. In the case of selected SDs, the n value was more than 0.89, indicating that CHS was released by matrix polymer diffusion and relaxation [36]. The Korsmeyer-Peppas remained the best kinetic model for SDs with poloxamer 407 and copovidone after 12 months of stability studies. Small changes in the release kinetics have occurred for SDs with macrogol 6000, where the R^2^ values were almost equal for the Higuchi and Korsmeyer-Peppas model (Table 4). The Higuchi model describes the release mechanism of APIs based on Fick’s diffusion and is commonly used to describe release kinetics for the modified-release dosage form [37].

**Table 4:** Release kinetic of dissolution data (initially and after 12 months of stability studies).

SD	0-order		I order		Korsmeyer-Peppas				Higuchi		Hixon-Crowell	
	R^2^		R^2^		R^2^		N		R^2^		R^2^	
	0m	12m	0m	12m	0m	12m	0m	12m	0m	12m	0m	12m
**P5**	0.6430	0.6419	0.8287	0.8240	0.9576	0.9628	1.8725	1.7098	0.8840	0.8830	0.7609	0.7575
**P9**	0.5450	0.7168	0.7126	0.9714	0.8433	0.9895	1.9614	1.6071	0.8113	0.9300	0.6397	0.8988
**M5**	0.6402	0.8102	0.8805	0.9094	0.9673	0.9713	1.8928	1.3559	0.8794	0.9714	0.7956	0.8787
**M9**	0.6573	0.7097	0.8869	0.7720	0.9970	0.9163	1.8761	1.2724	0.8915	0.9193	0.8057	0.7507
**C5**	0.6464	0.6602	0.7858	0.7858	0.9287	0.9840	1.8268	1.6257	0.8851	0.8952	0.7348	0.7434
**C9**	0.6338	0.6291	0.8342	0.8111	0.9863	0.9594	1.8867	1.7410	0.8751	0.8737	0.7619	0.7447

Clopidogrel content in the chosen SDs was in range of 95.98% to 99.86% after 12 months of stability studies (Table 3). Presented results for clopidogrel content were very similar compared to initial results and followed ICH Q1A (R2) guideline requirements [16].

DSC curves for all selected SDs after 12 months of stability studies (Figs 2–4) showed minimal changes in appearance and position of endothermic peaks, which confirmed that drug physical state was not changed during stability studies.

The FT-IR spectra of selected SDs after 12 months of stability studies (Figs 5–7) showed similar spectra with minimal or no differences in characteristic band positions and the absence of CHS crystallization in SDs samples which indicated that intermolecular interactions could stabilize the amorphous form of CHS in solid dispersions.

## 4. Conclusions

Initial results of the *in vitro* dissolution test showed that SDs increased the amount of dissolved CHS from all prepared samples compared to pure CHS, corresponding physical mixtures, and commercial tablet. The results obtained during the long-term stability study of selected clopidogrel hydrogen sulfate SDs (clopidogrel content, DSC, and FT-IR) indicated good stability of SDs prepared with poloxamer 407, macrogol 6000, and copovidone, at CHS/polymer ratios 1:5 and 1:9. However, the *in vitro* dissolution test showed a considerable reduction in CHS released from SDs with macrogol 6000. Results obtained indicated the great importance of the *in vitro* dissolution test in determining the long-term stability and quality of SDs. Further studies are needed to explain better the changes in SDs with macrogol 6000, which occur over time, leading to a considerable reduction in the CHS released amount from these samples.
